# Multivariate analysis of prognostic factors in patients with lung cancer

**DOI:** 10.3389/fonc.2023.1022862

**Published:** 2023-02-22

**Authors:** Changjiang Liu, Minting Ma, Xuetao Zhou, Zefeng Zhang, Yang Guo

**Affiliations:** ^1^ Department of Thoracic Surgery, The Fourth Hospital of Hebei Medical University, Shijiazhuang, China; ^2^ Department of Medical Oncology, The Fourth Hospital of Hebei Medical University, Shijiazhuang, China

**Keywords:** lung cancer, prognosis, multivariate analysis, age, sex

## Abstract

**Objective:**

Lung cancer is the leading cause of cancer-related mortality in China. The purpose of this study was to determine the effect of non-therapeutic and therapeutic factors of patients with lung cancer on survival rate.

**Methods:**

In this retrospective study, a total of 458 patients diagnosed as lung cancer at the Department of Thoracic Surgery, the Fourth Affiliated Hospital of Hebei Medical University from September 2008 to October 2013 were enrolled. The COX proportional hazards model was used to analyze the possible factors affecting the survival of patients. Model variables included age, sex, family history, smoking, tumor location, pathological type, stage, chemotherapy, radiotherapy, operation, and targeted therapy.

**Results:**

The median survival time (MST) was 32.0 months (95% CI: 29.0-34.0 months), while the 1-, 3-, and 5-year survival rates were 70.74%, 36.90%, and 30.13%, respectively. The univariate analysis showed that stage, chemotherapy, radiotherapy, and operation significantly affected the median survival time of patients. Multivariate cox regression analysis suggested that sex (female *vs* male, 2.096, 95% CI: 1.606-2.736), stage (stage I *vs* IV, 0.111, 95% CI: 0.039-0.314; stage II *vs* IV, 0.218, 95%CI: 0.089-0.535), chemotherapy (no *vs* yes, 0.469, 95% CI: 0.297-0.742), and operation (no *vs* yes, 2.667, 95% CI: 1.174-6.055) were independently associated with the survival of patients with lung cancer.

**Conclusion:**

Our study showed that male, early stage, operation were protective factors for the survival of patients, while female, advanced stage, chemotherapy were risk factors for the survival of patients. Larger studies are required to address the usefulness of these prognostic factors in defining the management of patients with lung cancer.

## Introduction

Lung cancer is a leading cause of cancer-related mortality around the world, with more than 1.8 million new cases and approximately 1.6 million deaths annually (1–3). In China, Lung cancer has replaced liver cancer as the most commonly diagnosed cancer and the primary cause of cancer-related death for both men and women (4–6). According to the National Central Cancer Registry (NCCR), there were more than 600’000 new lung cancer diagnoses in China in 2010, which accounts for 19.59% of all new cancer cases (7). Therefore, lung cancer has imposed an enormous burden on the patients and society.

It is widely accepted that lung cancer is a group of clinical entities that share cellular and molecular origins but that have various clinical behaviors which result in different prognosis (8–10). Thus, analyzing the prognosis for an individual patient with lung cancer is difficult. It is reported that the overall mortality rates for lung cancer over the last 15 years have remained stable in North America (11). Previous studies have shown that smoking, gender, age and TNM staging of lung cancer are the main prognostic basis for lung cancer (12). Knowledge of these prognostic factors allows us to choose a personalized treatment for an individual patient. The purpose of this study was to determine the effect of non-therapeutic and therapeutic factors of patients with lung cancer on their 5-year survival rates by univariate and multivariate analyses.

## Methods

### Patients

This retrospective study enrolled 458 Chinese patients who had pathologically diagnosed as lung cancer at the Department of Thoracic Surgery, the Fourth Affiliated Hospital of Hebei Medical University from September 2008 to October 2013.

Inclusion criteria: (1) patients with a age ≥18 years; (2) patients with histological diagnosis of lung cancer. Exclusion criteria:(1) patients with tumors of other systems; (2) Patients with incomplete clinical information; (3) Patients who lost to follow-up.

The primary output of this study includes age, sex, family history, smoking status, tumor location, tumor histopathological type, stage, and therapy. The endpoint variable in this study included patient mortality at one, three, and five years.

### The CT examination protocol

The Chest CT scan protocol: All patients underwent chest CT plain scan after routine chest X-ray examination. Hitachi CT(ECLOS) 16-slice CT machine was used for CT examination, and the parameters of the instrument scan examination were as follows: Current control is 50Ma, voltage control is 100Kv, layer spacing is 5mm, pitch is 1.25mm, layer spacing and reconstruction layer thickness is 5mm, scanning time is 1.53s.

### Statistical analysis

Statistical analysis was performed with SAS 9.3 (SAS Institute, Cary, NC, United State). Continuous variables satisfying normal distribution were described by means of standard deviation. The categorical data was presented as prevalence (%). Survival curves were analyzed using the Kaplan-Meier method and compared using the log-rank test to demonstrate the effects of different stages, radiotherapy, chemotherapy and surgery on the survival time of patients. Variables with statistically significant differences in univariate comparison were included in multivariate Cox regression. Multivariate Cox regression analysis was used to evaluate the independent prognostic factors of lung cancer. A cutoff P value of 0.05 was adopted for all analyses.

## Results

**Figure 1:**
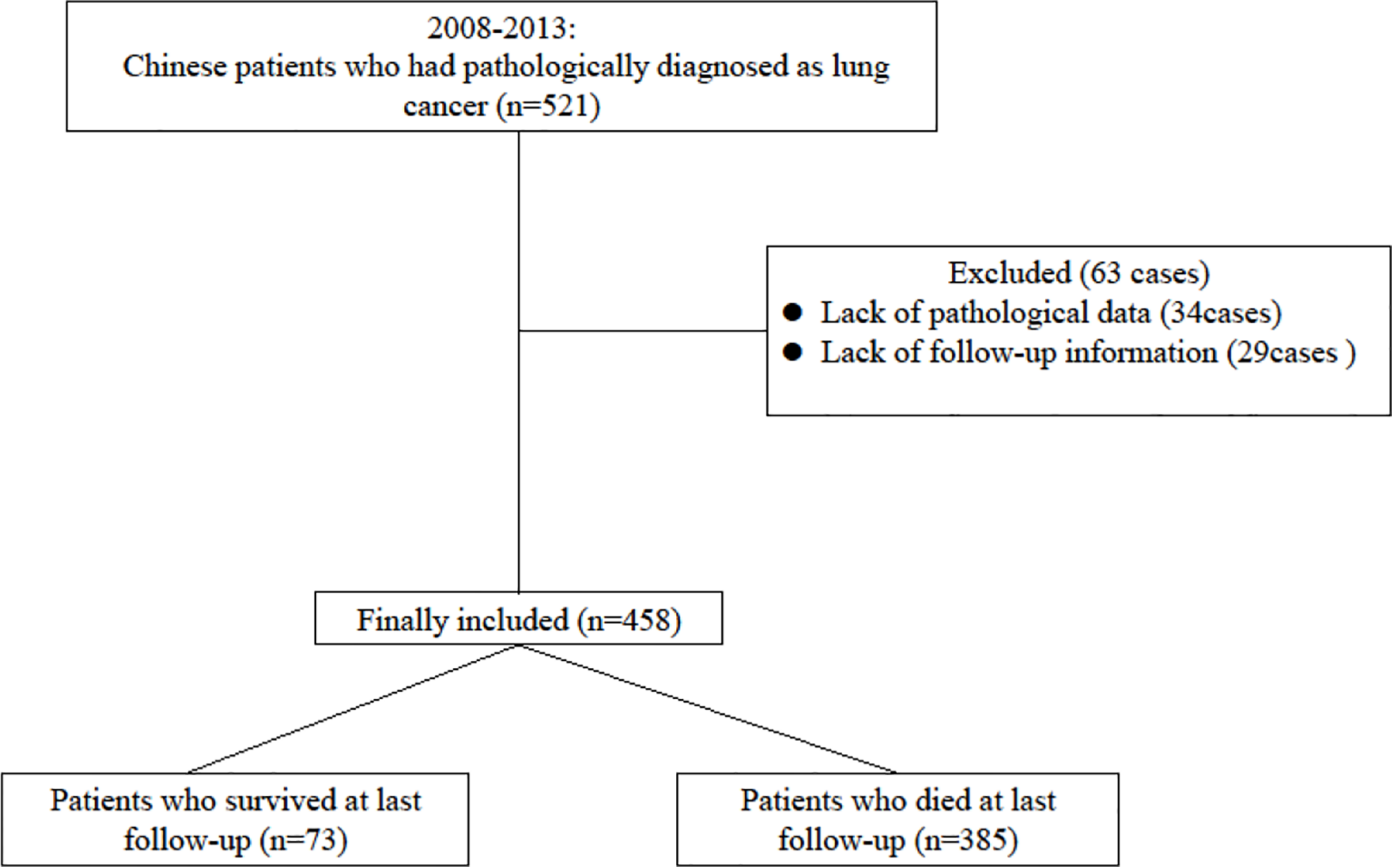
The flowchart of patient enrollment.

A total of 458 patients with lung cancer were enrolled in this study. During the five years of follow-up, 138 (30.13%) patients were survival, and 300 (65.50%) patients were dead, and 20 (4.37%) patients were lost of follow-up. The median survival time (MST) was 32.0 months (95% CI: 29.0-34.0 months), while the 1-, 3-, and 5-year survival rates were 70.74%, 36.90%, and 30.13%, respectively. The patient enrollment process was shown in **Figure 1**.

**Figure 2:**
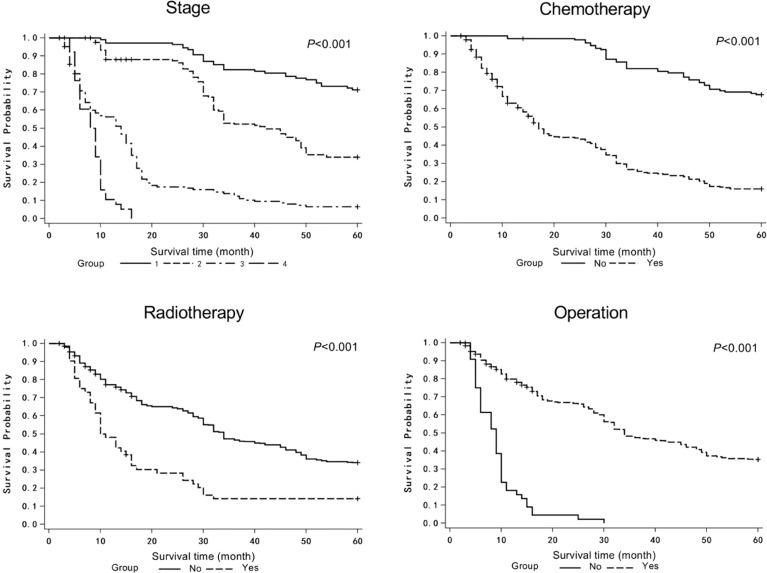
Survival curves for patients with lung cancer.

The COX proportional hazards model was used to analyze the possible factors affecting the survival of patients with lung cancer. Model variables included age, sex, family history, smoking, tumor location, pathological type, stage, chemotherapy, radiotherapy, operation, and targeted therapy (**Table 1**). The age of the patients was 59.54 ± 9.30 years, and 138 (30.13%) patients were female. Only 18 patients had family history, while 223 (48.69%) patients were smokers. The tumor locations included left upper lobe (n=86, 18.78%), left lower lobe (n=89, 19.43%), right upper lobe (n=92, 20.09%), right lower lobe (n=94, 20.52%), and others (n=97, 21.18%). The majority of histopathological type was squamous cell carcinoma (n=158, 34.50%) and adenocarcinoma (n=215, 46.94%). The tumor stages were as follows: stage I (n=108, 23.58%), stage II (n=163, 35.59%), stage III (n=148, 32.31%), and stage IV (n=49, 8.52%). Majority of patients underwent chemotherapy (n=324, 70.74%) and operation (n=413, 90.17%), while part of patients received radiotherapy (n=52, 11.35%) and targeted therapy (n=42, 9.17%). Univariate analysis showed that stage, chemotherapy, radiotherapy, and operation significantly affected the MST of patients (**Table 1** and **Figure 2**). In multivariate analysis, reported as hazard ratio and 95% CI, the result showed that sex (female *vs* male, 2.096, 95% CI: 1.606-2.736), stage (stage I *vs* IV, 0.111, 95% CI: 0.039-0.314; stage II *vs* IV, 0.218, 95%CI: 0.089-0.535), chemotherapy (no *vs* yes, 0.469, 95% CI: 0.297-0.742), and operation (no *vs* yes, 2.667, 95% CI: 1.174-6.055) were independently associated with the survival of patients with lung cancer (**Table 2**). These results indicated that male, early stage, operation were protective factors for the survival of patients, while female, advanced stage, chemotherapy were risk factors for the survival of patients.

**Table 1:** Univariate analysis of prognostic factors in lung cancer.

Factors	n (%)	Survival rate (%)			Mst (month)	95% CI (month)	*P* value
		1-year	3-year	5-year			
Total	458	70.74	36.90	30.13	32.0	29.0-34.0	
Age							0.090
y<55	129 (28.17)	67.44	30.23	21.71	28.0	17.0-32.0	
55≤y<60	88 (19.21)	69.32	36.36	36.36	32.0	26.0-48.0	
60≤y<65	99 (21.62)	74.75	40.40	31.31	32.0	26.0-48.0	
y≥65	142 (31.00)	71.83	40.85	33.10	34.0	29.0-49.0	
Sex							0.123
Female	138 (30.13)	52.90	36.96	27.54	28.0	11.0-34.0	
Male	320 (69.87)	78.44	36.88	31.25	32.0	30.0-34.0	
Family history							0.362
Yes	18 (3.93)	66.67	33.33	22.22	22.5	10.0-48.0	
No	440 (96.07)	70.91	37.05	30.45	32.0	29.0-34.0	
Smoking							0.497
Yes	223 (48.69)	79.37	38.12	31.84	32.0	29.0-37.0	
No	235 (51.31)	62.55	35.74	28.51	30.0	27.0-34.0	
Tumor location							0.146
Left upper lobe	86 (18.78)	90.70	47.67	36.05	30.0	18.0-42.0	
Left lower lobe	89 (19.43)	62.92	32.58	29.21	37.0	30.0-50.0	
Right upper lobe	92 (20.09)	70.65	38.04	29.35	34.0	26.0-46.0	
Right lower lobe	94 (20.52)	73.40	39.36	31.91	30.0	24.0-45.0	
Others	97 (21.18)	57.73	27.84	24.74	18.0	11.0-32.0	
Pathological type							0.273
Squamous cell carcinoma	158 (34.50)	78.48	41.14	32.91	34.0	30.0-45.0	
Adenocarcinoma	215 (46.94)	68.84	37.67	30.23	31.0	27.0-34.0	
Adenoacanthoma	30 (6.55)	70.00	36.67	30.00	28.0	17.0-54.0	
Others	55 (12.01)	56.36	21.82	21.82	19.0	11.0-34.0	
Stage							<0.001
I	108 (23.58)	97.22	82.41	71.30	–	–	
II	163 (35.59)	84.66	49.08	31.90	42.0	34.0-49.0	
III	148 (32.31)	52.03	6.08	0.00	14.0	9.0-15.0	
IV	39 (8.52)	10.26	0.00	0.00	8.5	6.0-9.0	
Chemotherapy							<0.001
Yes	324 (70.74)	59.26	18.52	14.81	17.0	16.0-21.0	
No	134 (29.26)	98.51	81.34	67.67	–	–	
Radiotherapy							<0.001
Yes	52 (11.35)	48.08	13.46	3.85	10.5	9.0-16.0	
No	406 (88.65)	73.65	41.13	32.27	34.0	31.0-40.0	
Operation							<0.001
Yes	413 (90.17)	76.51	40.92	33.41	34.0	32.0-42.0	
No	45 (9.83)	17.78	0.00	0.00	9.0	6.0-10.0	
Targeted therapy							0.357
Yes	42 (9.17)	64.29	28.57	26.19	27.0	10.0-48.0	
No	416 (90.83)	71.39	37.74	30.53	32.0	29.0-34.0	

MST, median survival time; CI, confidence interval.

**Table 2:** Multivariate analysis of survival factors in lung cancer by COX proportional hazards regression model.

Factors	Class	HR	95% CI	*P* value
Sex	female *vs* male	2.096	1.606-2.736	<0.001
Stage	1 *vs* 4	0.111	0.039-0.314	<0.001
Stage	2 *vs* 4	0.218	0.089-0.535	0.001
Chemotherapy	no *vs* yes	0.469	0.297-0.742	0.001
Operation	no *vs* yes	2.667	1.174-6.055	0.019
Targeted therapy	no *vs* yes	1.462	0.998-2.141	0.051

HR, hazard ratio; CI, confidence interval.

## Discussion

Lung cancer is the leading cause of cancer-related death in China with an extremely poor overall survival rate. In this study, by analyzing our data on 458 patients using a Cox multivariable regression model, we identified sex, stage, chemotherapy, and operation as the independent prognostic factors for lung cancer.

We found two non-therapeutic factors, including stage and sex, which were the independent prognostic factors for lung cancer. Lung cancer is generally staged by the TNM staging system. Consistent with previous studies (11–14), our data revealed that stage is the most powerful prognostic tool for predicting the survival rates of patients with lung cancer. Numerous studies have reported that female patients have better relative survival compared with male patients for each stage of disease (15, 16). The increase in survival may attribute to the increased life expectancy of women relative to men, or the inherent biological differences between the sexes. However, our study showed that female patients have lower survival rate than male patients, especially in the first year of follow-up. The reason behind this phenomenon remains unknown.

In addition, we found two therapeutic factors, including chemotherapy and operation, which were the independent prognostic factors for lung cancer. As for adjuvant therapy, the NationalComprehensive Cancer Network (NCCN) recommends postoperative chemotherapy for patients with N2 NSCLC, and the main chemotherapy regimen is mainly platinum drugs supplemented with vincrine or pemetrexed (17, 18). In a large population-based study, adjuvant radiotherapy in patients with stage N2 improved patient outcomes (19). Surgery still remains the single most consistent and successful option for cure for patients with lung cancer (20). Our data showed that patients with operation have higher survival rate than patients without operation. This may be because lymph nodes can be dissected more systematically during surgery, and obtaining more accurate lymph node metastases is of great value to improve the prognosis of patients. Unfortunately, the disease is always diagnosed at a stage too advanced to allow this treatment for a majority of patients. For patients with locally advanced and metastatic disease, chemotherapy is beneficial for palliation. However, our data revealed that the subgroup of patients received chemotherapy experienced lower survival rates. Despite advances in standard therapies, survival rate remains poor for patients with lung cancer.

Increased understanding of cancer biology has revealed several effective therapeutic strategies, including targeted therapy (21–23). Over the last decade, targeted therapy has become firmly established as a therapeutic option in lung cancer. Our study found that, though not statistically significant (*P*=0.051), patients received targeted therapy seem to have better prognosis. Epidermal growth factor receptor (EGFR) somatic mutations are the most common type of mutation in NSCLC Non-Smallcell lung cancer (NSCLC). Patients with this group of mutations are more likely to receive treatment of epidermal growth factor receptor-tyrosinekinase inhibitor (EGFR-TKIs), because of its high efficiency and low toxicity. High objective response rate. These patients have a significantly longer life expectancy than patients who were treated with chemotherapy alone in the past (24–26). In addition, early screening is an independent prognostic factor that can benefit patients with lung cancer in order to improve survival. Previous reports suggest that overall mortality is significantly higher in the screening group than in the non-screening group, and highlight the benefits of low-dose computed tomography (LDCT) screening programs in Asian populations with a high incidence of non-smoking-related lung cancer (27–29).

There still have some limitations in the present study. Firstly, due to the retrospective nature of this study, the patients were from a single center, and the sample size was relatively limited. Secondly, the current patients were not followed up for long enough, nor were subgroup analyses between sexes sufficiently performed. Therefore, further studies with more scientific sample size and more comprehensive design are still needed.

In summary, our data revealed that sex, stage, chemotherapy, and operation are the independent prognostic factors for lung cancer. Larger studies are required to address the usefulness of these prognostic factors in defining the management of patients with lung cancer.

## Data availability statement

The original contributions presented in the study are included in the article/Supplementary Material. Further inquiries can be directed to the corresponding author.

## Ethics statement

The studies involving human participants were reviewed and approved by The Fourth Hospital of Hebei Medical University. The patients/participants provided their written informed consent to participate in this study.

## Author contributions

CL and YG contributed to the conception and design of the study. MM, XZ, and ZZ performed the experiments, collected and analyzed data. CL and YG wrote the manuscript. All authors contributed to the article and approved the submitted version.

## References

[1] Jamal-Hanjani M Wilson GA McGranahan N Birkbak NJ Watkins TB K Veeriah S. Tracking the evolution of non-small-cell lung cancer. N Engl J Med (2017) 376(22):2109–21. doi: 10.1056/NEJMoa1616288 28445112

[2] Hirsch FR Scagliotti GV Mulshine JL Kwon R Curran W J Jr Wu YL. Lung cancer: Current therapies and new targeted treatments. Lancet (2017) 389(10066):299–311. doi: 10.1016/S0140-6736(16)30958-8 27574741

[3] Ettinger DS Wood DE Akerley W Bazhenova LA Borghaei H Camidge DR. Non-small cell lung cancer, version 6. 2015. J Natl Compr Canc Netw (2015) 13(5):515–24. doi: 10.6004/jnccn.2015.0071 25964637

[4] Hong QY Wu GM Qian GS Hu CP Zhou JY Chen LA. Prevention and management of lung cancer in China. Cancer (2015) 121 Suppl 17:3080–8. doi: 10.1002/cncr.29584 26331814

[5] Chen W Zheng R Zeng H Zhang SW He J. Annual report on status of cancer in China, 2011. Chin J Cancer Res (2015) 27(1):2–12. doi: 10.1186/s40880-015-0001-2 25717220 PMC4329176

[6] Zheng R Zeng H Zhang S Chen TH Chen WQ. National estimates of cancer prevalence in China, 2011. Cancer Lett (2016) 370(1):33–8. doi: 10.1016/j.canlet.2015.10.003 26458996

[7] Cao M Chen W. Epidemiology of lung cancer in China. Thorac Cancer. (2019) 10(1):3–7. doi: 10.1111/1759-7714.12916 30485694 PMC6312841

[8] Saito M Shiraishi K Kunitoh H Takenoshita S Yokota J Kohno T. Gene aberrations for precision medicine against lung adenocarcinoma. Cancer Sci (2016) 107(6):713–20. doi: 10.1111/cas.12941 PMC4968599 27027665

[9] Sun S Schiller JH Gazdar AF. Lung cancer in never smokers–a different disease. Nat Rev Cancer. (2007) 7(10):778–90. doi: 10.1038/nrc2190 17882278

[10] Tomasetti C Li L Vogelstein B. Stem cell divisions, somatic mutations, cancer etiology, and cancer prevention. Science (2017) 355(6331):1330–4. doi: 10.1126/science.aaf9011 PMC5852673 28336671

[11] Brundage MD Davies D Mackillop WJ. Prognostic factors in non-small cell lung cancer: a decade of progress. Chest (2002) 122(3):1037–57. doi: 10.1378/chest.122.3.1037 12226051

[12] Atci MM Sakin A Uysal E Aksaray F Selvi O Can O. Survival and prognostic factors in limited-stage small-cell lung cancer. J Coll Physicians Surg Pak (2021) 31(12):1433–7. doi: 10.29271/jcpsp.2021.12.1433 34794283

[13] Paesmans M Sculier JP Libert P Bureau G Dabouis G Thiriaux J. Prognostic factors for survival in advanced non-small-cell lung cancer: Univariate and multivariate analyses including recursive partitioning and amalgamation algorithms in 1,052 patients. The European lung cancer working party. J Clin Oncol (1995) 13(5):1221–30. doi: 10.1200/JCO.1995.13.5.1221 7738625

[14] Spiegelman D Maurer LH Ware JH Perry MC Chahinian AP Comis R. Prognostic factors in small-cell carcinoma of the lung: An analysis of 1,521 patients. J Clin Oncol (1989) 7(3):344–54. doi: 10.1200/JCO.1989.7.3.344 2537384

[15] Kiyohara C Ohno Y. Sex differences in lung cancer susceptibility: A review. Gend Med (2010) 7(5):381–401. doi: 10.1016/j.genm.2010.10.002 21056866

[16] Wakelee HA Gomez SL Chang ET. Sex differences in lung-cancer susceptibility: A smoke screen? Lancet Oncol (2008) 9(7):609–10. doi: 10.1016/S1470-2045(08)70162-1 PMC6393927 18598927

[17] Ettinger DS Aisner DL Wood DE Akerley W Bauman J Chang JY. NCCN guidelines insights: Non-small cell lung cancer, version 5. 2018. J Natl Compr Canc Netw (2018) 16(7):807–21. doi: 10.6004/jnccn.2018.0062 30006423

[18] Arriagada R Bergman B Dunant A Chevalier TL Pignon JP Vansteenkiste J. Cisplatin-based adjuvant chemotherapy in patients with completely resected non-small-cell lung cancer. N Engl J Med (2004) 350(4):351–60. doi: 10.1056/NEJMoa031644 14736927

[19] Lally BE Zelterman D Colasanto JM Haffty BG Detterbeck FC Wilson LD. Postoperative radiotherapy for stage II or III non-small-cell lung cancer using the surveillance, epidemiology, and end results database. J Clin Oncol (2006) 24(19):2998–3006. doi: 10.1200/JCO.2005.04.6110 16769986

[20] Molina JR Yang P Cassivi SD Schild SE Adjei AA. Non-small cell lung cancer: epidemiology, risk factors, treatment, and survivorship. Mayo Clin Proc (2008) 83(5):584–94. doi: 10.1016/S0025-6196(11)60735-0 PMC2718421 18452692

[21] Chan BA Hughes BG. Targeted therapy for non-small cell lung cancer: current standards and the promise of the future. Transl Lung Cancer Res (2015) 4(1):36–54. doi: 10.3978/j.issn.2218-6751.2014.05.01 25806345 PMC4367711

[22] Herbst RS Morgensztern D Boshoff C. The biology and management of non-small cell lung cancer. Nature (2018) 553(7689):446–54. doi: 10.1038/nature25183 29364287

[23] Sequist LV Soria JC Goldman JW Wakelee HA Gadgeel SM Varga A. Rociletinib in EGFR-mutated non-small-cell lung cancer. N Engl J Med (2015) 372(18):1700–9. doi: 10.1056/NEJMoa1413654 25923550

[24] Wu YL Cheng Y Zhou X Lee KH Nakagawa K Niho SJ. Dacomitinib versus gefitinib as first-line treatment for patients with EGFR-mutation-positive non-small-cell lung cancer (ARCHER 1050): a randomised, open-label, phase 3 trial. Lancet Oncol (2017) 18(11):1454–66. doi: 10.1016/S1470-2045(17)30608-3 28958502

[25] Park K Tan EH O'Byrne K Zhang L Boyer M Mok T. Afatinib versus gefitinib as first-line treatment of patients with EGFR mutation-positive non-small-cell lung cancer (LUX-lung 7): a phase 2B, open-label, randomised controlled trial. Lancet Oncol (2016) 17(5):577–89. doi: 10.1016/S1470-2045(16)30033-X 27083334

[26] Wu YL Zhou C Hu CP Feng JF Lu S Huang YC. Afatinib versus cisplatin plus gemcitabine for first-line treatment of Asian patients with advanced non-small-cell lung cancer harbouring EGFR mutations (LUX-lung 6): An open-label, randomised phase 3 trial. Lancet Oncol (2014) 15(2):213–22. doi: 10.1016/S1470-2045(13)70604-1 24439929

[27] Wu FZ Kuo PL Huang YL Tang EK Chen CS Wu MT. Differences in lung cancer characteristics and mortality rate between screened and non-screened cohorts. Sci Rep (2019) 9(1):19386. doi: 10.1038/s41598-019-56025-6 31852960 PMC6920422

[28] Wu FZ Wu YJ Chen CS Yang SC. Impact of smoking status on lung cancer characteristics and mortality rates between screened and non-screened lung cancer cohorts: Real-world knowledge translation and education. J Pers Med (2022) 12(1):26. doi: 10.3390/jpm12010026 35055341 PMC8780024

[29] Wu FZ Huang YL Wu CC Tang EK Chi-Shen Chen CS Mar GY. Assessment of selection criteria for low-dose lung screening CT among Asian ethnic groups in Taiwan: From mass screening to specific risk-based screening for non-smoker lung cancer. Clin Lung Cancer. (2016) 17(5):e45–56. doi: 10.1016/j.cllc.2016.03.004 27133540

